# Stimulatory effects of the degradation products from Mg-Ca-Sr alloy on the osteogenesis through regulating ERK signaling pathway

**DOI:** 10.1038/srep32323

**Published:** 2016-09-01

**Authors:** Mei Li, Peng He, Yuanhao Wu, Yu Zhang, Hong Xia, Yufeng Zheng, Yong Han

**Affiliations:** 1 State Key Laboratory for Mechanical Behavior of Materials, Xi’an Jiaotong University, Xi’an 710049, China; 2 Department of Orthopedics, Nanjing General Hospital of Nanjing Military Command, 305 zhongshandong road, Nanjing 210002, China; 3 Center for Biomedical Materials and Tissue Engineering, Academy for Advanced Interdisciplinary Studies, Peking University, Beijing 100871, China; 4 Department of Orthopedics, Guangdong Key Lab of Orthopaedic Technology and Implant Materials, Guangzhou General Hospital of Guangzhou military command, 111 Liuhua Road, Guangzhou 510010, China; 5 Department of Materials Science and Engineering, College of Engineering, Peking University, Beijing 100871, China

## Abstract

The influence of Mg-1Ca-xwt.% Sr (x = 0.2, 0.5, 1.0, 2.0) alloys on the osteogenic differentiation and mineralization of pre-osteoblast MC3T3-E1 were studied through typical differentiation markers, such as intracellular alkaline phosphatase (ALP) activity, extracellular collagen secretion and calcium nodule formation. It was shown that Mg-1Ca alloys with different content of Sr promoted cell viability and enhanced the differentiation and mineralization levels of osteoblasts, and Mg-1Ca-2.0Sr alloy had the most remarkable and significant effect among all. To further investigate the underlying mechanisms, RT-PCR and Western Blotting assays were taken to analyze the mRNA expression level of osteogenesis-related genes and intracellular signaling pathways involved in osteogenesis, respectively. RT-PCR results showed that Mg-1Ca-2.0Sr alloy significantly up-regulated the expressions of the transcription factors of Runt-related transcription factor 2 (RUNX2) and Osterix (OSX), Integrin subunits, as well as alkaline phosphatase (ALP), Bone sialoprotein (BSP), Collagen I (COL I), Osteocalcin (OCN) and Osteopontin (OPN). Western Blotting results suggested that Mg-1Ca-2.0Sr alloy rapidly induced extracellular signal-regulated kinase (ERK) activation but showed no obvious effects on c-Jun N terminal kinase (JNK) and p38 kinase of MAPK. Taken together, our results demonstrated that Mg-1Ca-2.0Sr alloy had excellent biocompatibility and osteogenesis via the ERK pathway and is expected to be promising as orthopedic implants and bone repair materials.

Mg-based biodegradable materials have garnered increasing attention for their potential applications as biodegradable and bioabsorbable implant materials in the field of orthopedics. This is primarily due to their attractive properties such as biocompatibility, low density, high specific strength and the ability to degrade and safely be absorbed under physiological conditions^1^,^2^,^3^, as well as can be better visualized in CT scans^4^. Unfortunately, the initial high degradation rate is accompanied by hydrogen evolution and solution alkalization which can delay the healing and cause implant loosening^5^. Alloying is a general and effective method to improve the corrosion resistance as well as the mechanical properties of Mg, and considerable efforts have focused on this approach, such as the addition of aluminium (Al), zirconium (Zr), praseodymium (Pr), yttrium (Y) and cerium (Ce) element^6^,^7^,^8^. However, there is still a potential hazard to the human body using some alloying elements, for example, the hepatotoxicity of Ce, Y and Pr at high concentrations^9^ and the neurotoxicity of Al. As a neurotoxicant, the accumulation of Al is associated with various neurological disorders, like dementia, senile dementia and Alzheimer disease^10^,^11^.

Strontium (Sr) is chemically and physically close to Calcium (Ca) and is a trace metal element in human bone. The potential to promote osteoblasts growth and diminish bone resorption of Sr has been demonstrated^12^. Besides that, small amounts of Sr could promote grain refinement, thereby improving the mechanical properties of pure Mg^13^,^14^. Therefore, Mg-Sr alloys have been recently evaluated as possible biodegradable materials for orthopedic implants^15^,^16^. Ca is the most abundant metal element in human body and the formation of calcium phosphates during the degradation process would provide more suitable local environment for bone mineralization^17^. Our previous study indicated that Mg alloy with 1 wt.% Ca content showed good mechanical properties, corrosion properties and biocompatibility *in vitro*, as well as bone regeneration effects *in vivo*^18^, therefore Mg-Ca alloy system has been proposed as another potential new kind of degradable metallic biomaterial with possible applications in bone tissues^15^,^19^. Berglund, Ida S. *et al.* has synthesized and characterized Mg-xCa-xSr (x = 0.5–7.0 wt%; y = 0.5–3.5 wt%) alloys for biodegradable orthopedic implant applications, and indicated that the Mg-1.0Ca-0.5Sr alloy is the most promising alloy since it showed the lowest degradation rate along with no significant toxicity to osteoblasts^20^, however, the blood compatibility, cyto-compatibility and the osteoinductivity as well as the corresponding mechanisms were not involved completely.

These beneficial effects of Ca and Sr prompted us to research the feasibility of Ca, Sr alloying within Mg and the corresponding effects on the biology functions. The purposes of our present study were: (1) to prepare Mg-1Ca alloys with different Sr contents; (2) to seek the optimal amount of Sr content added in Mg-1Ca alloys with respect to the osteogenic induction effects; (3) to illuminate the potential molecular mechanism of osteogenic differentiation about the Mg alloys with Ca and Sr.

## Results

**Figure 1:**
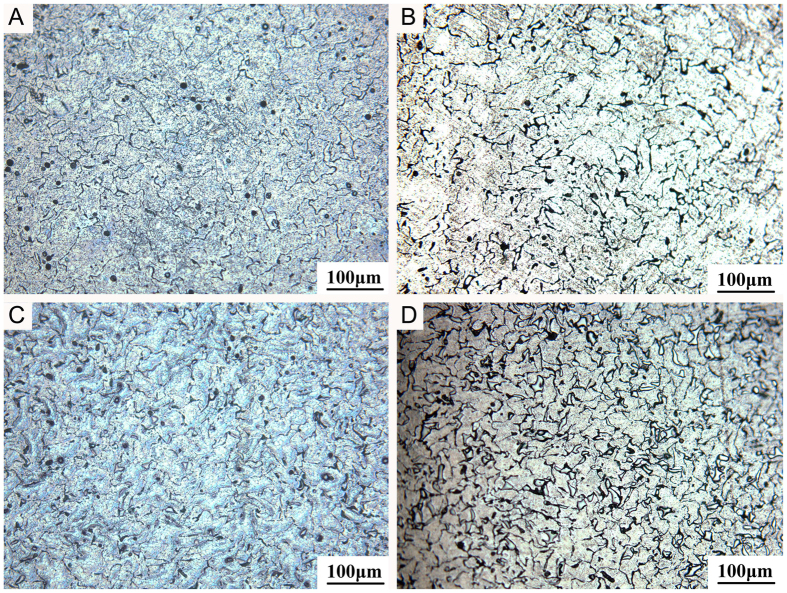
Optical micrographs of (**A**) Mg-1Ca-0.2Sr alloy; (**B**) Mg-1Ca-0.5Sr alloy; (**C**) Mg-1Ca-1.0Sr alloy; (**D**) Mg-1Ca-2.0Sr alloy samples.

**Figure 2:**
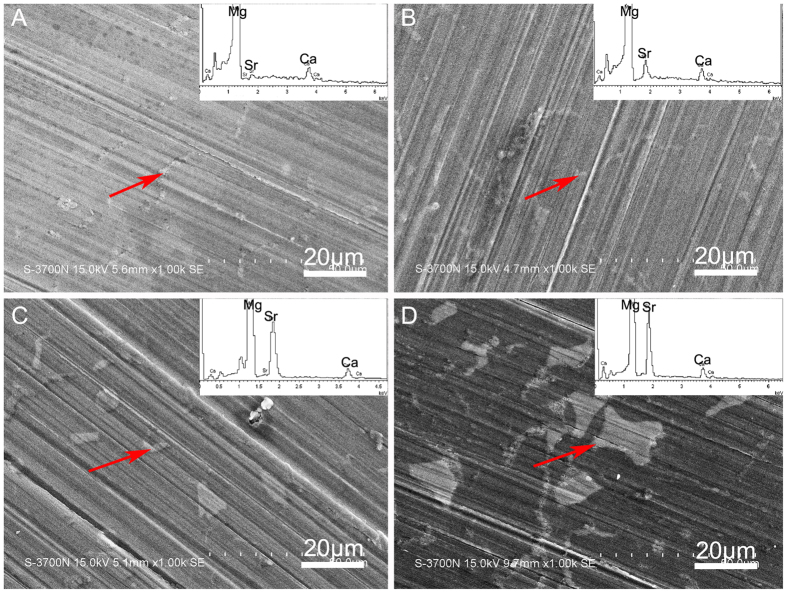
SEM micrograph and EDS analysis of (**A**) Mg-1Ca-0.2Sr alloy; (**B**) Mg-1Ca-0.5Sr alloy; (**C**) Mg-1Ca-1.0Sr alloy; (**D**) Mg-1Ca-2.0Sr alloy samples. The white particles and eutectic structure are arrowed. The inserts in upper right corner of A-D were corresponding EDS results of the red arrowed structure.

**Figure 3:**
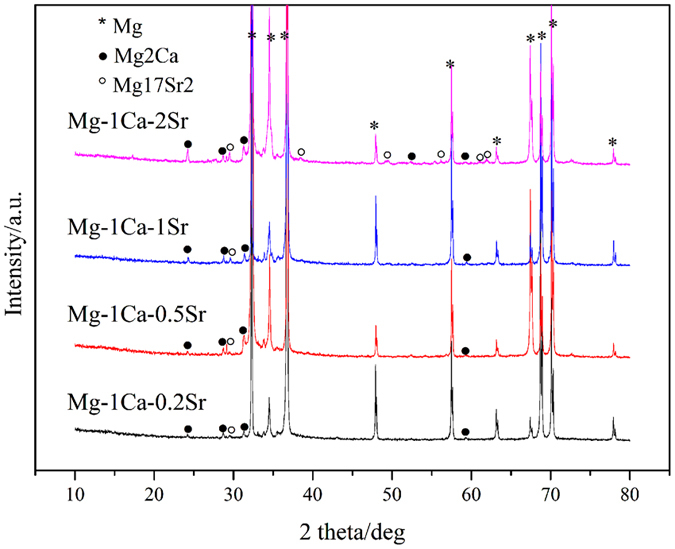
XRD patterns detected on the ternary Mg-1Ca-xwt.% Sr alloy (x = 0.2, 0.5, 1.0, 2.0) samples at room temperature. All four alloys displayed the same phases: α-Mg, Mg_2_Ca and Mg_17_Sr_2_.

Characterization of Mg alloys. The actual compositions of the alloys were determined by ICP-AES analysis and listed in Table 1. The optical micrographs of the ternary Mg-1Ca-xwt.% Sr (x = 0.2, 0.5, 1.0, 2.0) alloys were showed in Fig. 1, from which it can be seen that with the increasing Sr content, the grain size of the Mg alloys became more fine, indicating that the addition of Sr could promote the grain refinement. SEM and EDS (Fig. 2) revealed that the grain boundaries of the Mg-1Ca-xwt.% Sr alloys contained higher amounts of Sr and Ca, indicating the precipitate segregation of secondary phases during solidification, a common phenomenon prevalent in casting. With further increasing Sr content, more and coarser second phase particles were also presented in these alloys, and the addition of 2.0% Sr resulted in the grain sizes being 20 μm approximately (Fig. 2D). The phase identification of Mg-1Ca-x wt.% Sr alloys were characterized by XRD (Fig. 3), and the results showed that all alloys were composed of α-Mg phase, α-Mg_2_Ca and α-Mg_17_Sr_2_ intermetallic phase. It was also noticeable that the volume fraction of the Mg_17_Sr_2_ intermetallic phase was increased with the increasing Sr contents as shown in diffraction intensity.

### Protein adsorption assay and Hemocompatibility

**Figure 4:**
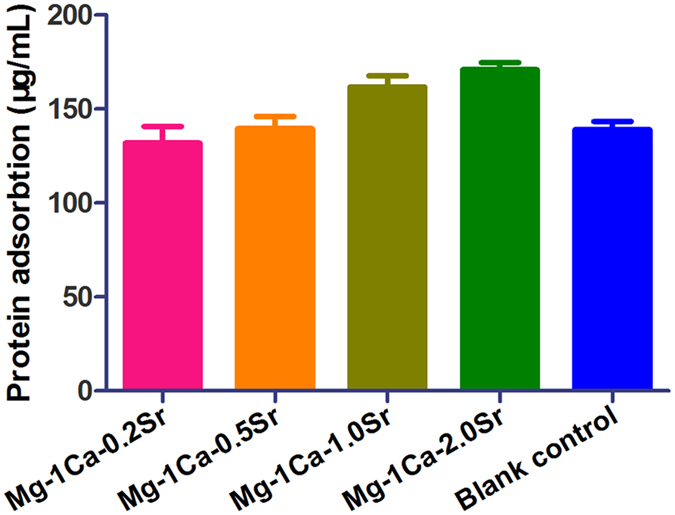
The quantity of protein absorption on four alloy samples after 4h of incubation in α-MEM medium containing 10% FBS. Mg-1Ca-2.0Sr alloy absorbed more protein than other three alloy specimens and blank control, however, the differences were not significant (p > 0.05).

A BCA protein assay kit was introduced to measure the quantity of protein adsorbed on the surface of different specimens from FBS and the results were shown in Fig. 4. It demonstrated that Mg-1Ca-0.2Sr alloy absorbed more protein than other three alloys, however the differences were not significant between different groups (p > 0.05).

The hemolytic test was carried out to assess the blood compatibility of different alloys and the results were summarized in Table 2. The HR value of Mg-1Ca-xwt.% (x = 0.2, 0.5, 1.0, 2.0) alloys was 0.21%, 1.48%, 1.05%, 2.01%, respectively. According to ISO 10993-4 standard, HR of the materials used in blood environment must be less than 5%. Therefore, our results indicated that all the four alloys had good hemocompatibility.

### Cell viability

**Figure 5:**
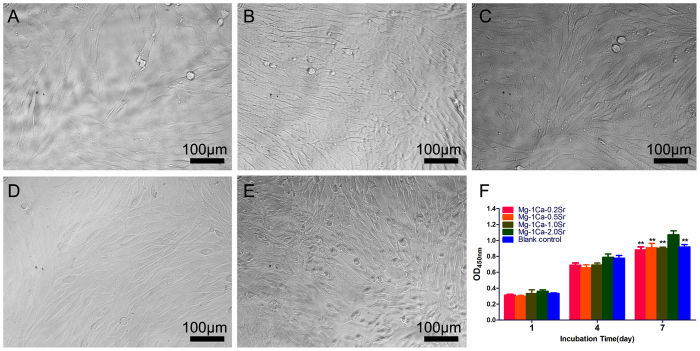
Cell viability assay in four alloys extracts after 1, 4 and 7 days incubation was measured by colorimetric CCK-8 assay. Cells cultured in α-MEM medium containing 10% FBS were set as control group. Cell morphology was observed by inverted phase contrast microscope on day 4, and the images showed that cells in different extracts were normal and healthy (**A**) Mg-1Ca-0.2Sr alloy; (**B**) Mg-1Ca-0.5Sr alloy; (**C**) Mg-1Ca-1.0Sr alloy; (**D**) Mg-1Ca-2.0Sr alloy), similar to that of the blank control (**E**). CCK-8 assay (**F**) showed that the absorbance of cells cultured in four Mg alloys extracts medium was gradually increased. At day 7, cell number of Mg-1Ca-2.0Sr alloy group was slightly higher than other group and reached the peak (p < 0.01). All data represent the mean ± standard deviation of three independent experiments. **p < 0.01 compared with Mg-1Ca-2.0Sr alloy group.

To investigate the effects of Ca, Sr, and Mg ions released from Mg-1Ca-xwt.% Sr alloy on the proliferation of pre-osteoblasts, cells were cultured in different extracts for 1, 4 and 7 days, respectively. Figure 5A–D showed the morphologies of MC3T3-E1 cells cultured in different alloys extracts after 4 days incubation. It can be seen that the cell morphologies were normal and healthy, similar to that of the control group (Fig. 5E). As determined by CCK-8 assay, Fig. 5F showed that cells cultured in four alloys extracts medium had relatively similar absorbance in comparison to the control group at day 1 (p > 0.05). Afterward, the absorbance value of all the groups was increased drastically, meaning that the number of living cells was increased. At day 7, the cell number of Mg-1Ca-2.0Sr alloy group was slightly higher than other groups (p < 0.01). Meanwhile, the absorbance value of other three alloy groups had no statistically significant differences compared with the control group (p > 0.05).

### Intracellular total protein content and ALP activity

**Figure 6:**
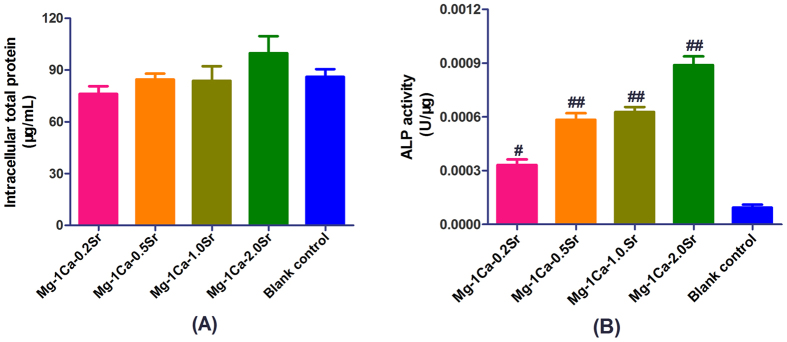
Intracellular total protein synthesis (**A**) and ALP activity (**B**) of osteoblasts osteo-induced by different alloy extracts 14 days of incubation. Cells cultured in osteogenic medium (α-MEM medium with 10% FBS, 0.01 μM dexamethasone, 50 ug/mL ascorbic acid and 10 mM Na-β-glycerophosphate) were set as the blank control. The ALP activity of four Mg alloys group were elevated and significantly higher than the blank control (p < 0.05), and Mg-1Ca-2.0Sr reached the highest value. All data represent the mean ± standard deviation of three independent experiments. ^#^p < 0.05 and ^##^p < 0.01 compared with the blank control group.

To investigate the effect of different extracts on osteogenic differentiation of pre-osteoblasts, the activity of ALP, as a representative marker of early stage of osteogenesis, was evaluated at 14 days after osteogenic induction. The intracellular total protein synthesis was measured simultaneously and results were showed in Fig. 6. The MC3T3-E1 cells cultured in four alloys extracts medium exhibited higher ALP activities than the control group (p < 0.05), and the Mg-1Ca-2.0Sr group had the highest value (p < 0.01). The order from high to low of the ALP activity level was: Mg-1Ca-2.0Sr > Mg-1Ca-1.0Sr > Mg-1Ca-0.5Sr > Mg-1Ca-0.2Sr.

### Collagen secretion

**Figure 7:**
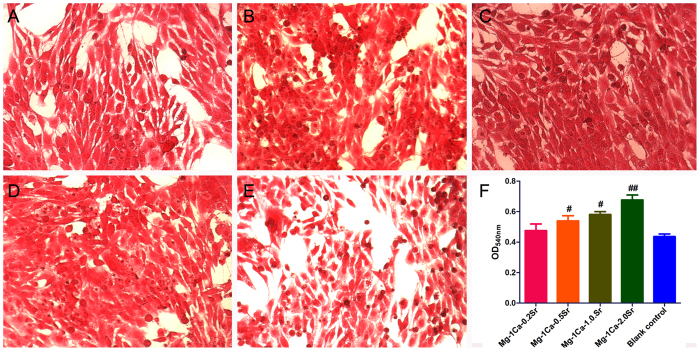
The collagen secretion was stained by Sirius Red (**A**) Mg-1Ca-0.2Sr alloy, (**B**) Mg-1Ca-0.5Sr alloy, (**C**) Mg-1Ca-1.0Sr alloy, (**D**) Mg-1Ca-2.0Sr alloy, (**E**) Control group) and quantified using a colorimetric method (**F**) after osteoinduction for 14 days. Cells cultured in osteogenic medium were set as the blank control group. All data represent the mean ± standard deviation of three independent experiments. ^**#**^p < 0.05 and ^**##**^p < 0.01 compared with the blank control group.

Collagen secretion was observed and quantified by the Sirius Red staining. Figure 7A–D showed that more collagen was secreted by Mg-1Ca-xwt.% Sr alloys compared to the blank control group (Fig. 7E), and obviously denser collagen induced by Mg-1Ca-2.0Sr alloy was deposited than other three alloys and blank control. According to the quantitative results as shown in Fig. 7F, collagen deposition inducing by Mg-1Ca-xwt.% (x = 0.2, 0.5, 1.0, 2.0) alloys promoted to about 108%, 125%, 121% and 155% compared to the control group, respectively.

### Extracellular matrix mineralization

**Figure 8:**
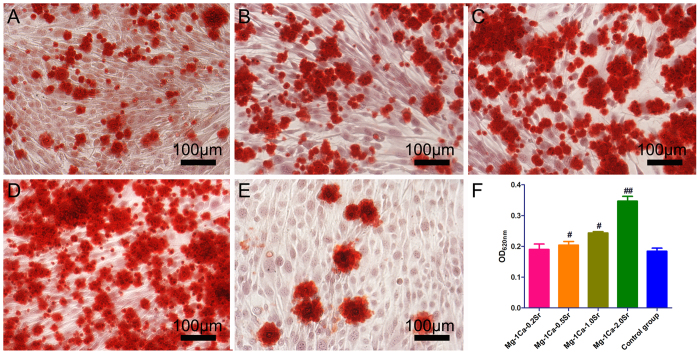
The extracellular calcium deposition was stained by Alizarin Red S (**A**) Mg-1Ca-0.2Sr alloy, (**B**) Mg-1Ca-0.5Sr alloy, (**C**) Mg-1Ca-1.0Sr alloy, (**D**) Mg-1Ca-2.0Sr alloy, (**E**) Control group) and quantified using a colorimetric method (**F**) after osteoinduction for 14 days. Cells cultured in osteogenic medium were set as the control group. With the increase of Sr content in four alloys, extracellular matrix mineralization nodules were stained intensively and increased visibly. All data represent the mean ± standard deviation of three independent experiments. ^**#**^p < 0.05 and ^**##**^p < 0.01 compared with the blank control group.

Calcium deposition, as a marker of the late stage of osteogenesis, was measured by Alizarin Red staining and showed in Fig. 8. With the increase of Sr content in Mg-1Ca alloys, extracellular matrix mineralization nodules were stained intensively and increased visibly. According to further quantitative results as shown in Fig. 8F, the relative value of matrix mineralization cultured with Mg-1Ca-xwt.% (x = 0.2, 0.5, 1.0, 2.0) alloys extract significantly enhanced to about 102%, 111%, 132% and 188% compared to the control group, respectively.

### Gene expression

**Figure 9:**
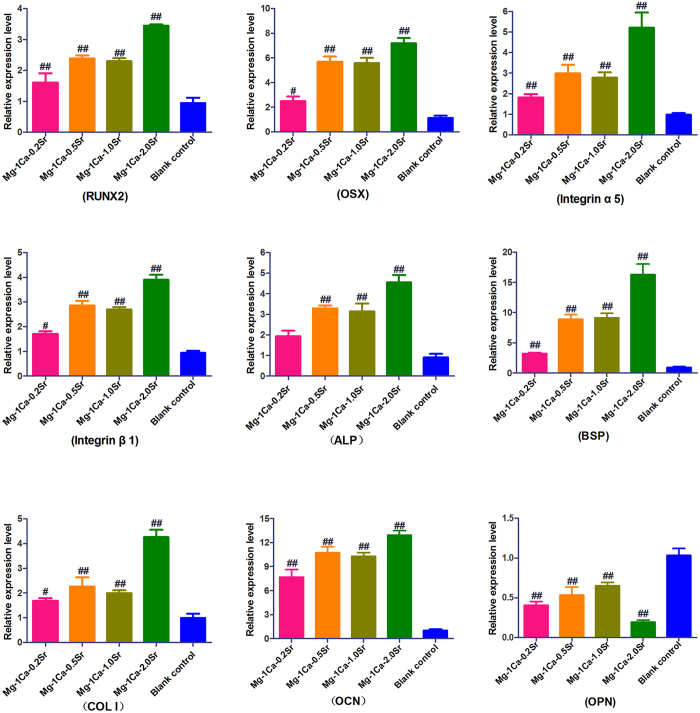
Relative expression level of top 9 differentially expressed genes in osteoblasts cultured with four alloy extracts for 14 days using SYBR Green RT-PCR. In each case, the data were normalized to the expression level of GAPDH as a housekeeping gene. Cells cultured in osteogenic medium were set as control. All data represent the mean ± standard deviation of three independent experiments. ^**#**^p < 0.05 and ^**##**^p < 0.01 compared with the control group.

The mRNA expression level of osteogenesis-related genes and integrin subunits after cultured in the alloys extracts medium was shown in Fig. 9. After 14 days of incubation, the expression level of RUNX2, OSX, Integrin α5, Integrin β1, ALP, BSP, COL I and OCN genes in Mg-1Ca-xwt.% Sr alloys was significantly higher than the control group (Fig. 9), however, the expression level of OPN gene was remarkably lower than the control group as shown in Fig. 9. Besides that, Mg-1Ca-2.0Sr alloy presented the most significant effects on the expression of osteogenesis-related genes including RUNX2, OSX, ALP, BSP, COL I and OCN (p < 0.01) except OPN.

### Phosphorylation of ERK1/2, JNK and p38 of MAPK signal pathway

**Figure 10:**
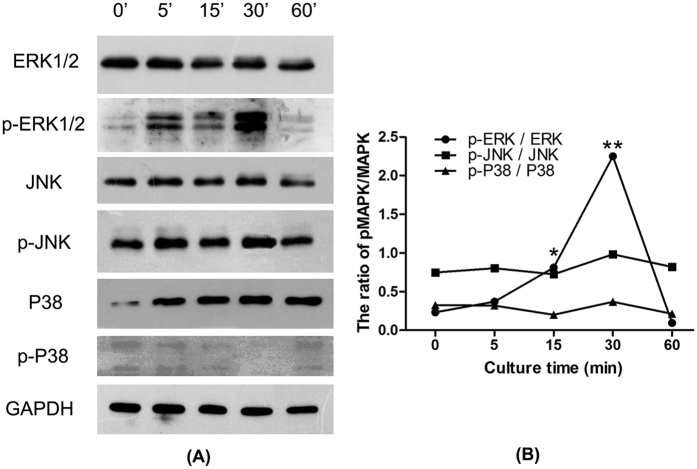
Activation of ERK in the osteogenic differentiation of osteoblasts cultured in four alloy extracts for 14 days. Following serum starvation for 24 h, MC3T3-E1 cells were treated with four serum-free alloy extracts for 0, 5, 15, 30 and 60 min, cells cultured in osteogenic medium were set as control. All data represent the mean ± standard deviation of three independent experiments. *p < 0.05 and **p < 0.01 compared with the 0 min.

Since Mg-1Ca with 2.0% content Sr was the most effective alloy to induce the osteogenic differentiation and mineralization of osteoblasts shown by the above results, we selected Mg-1Ca-2.0Sr alloy to investigate the possible signaling pathway. Numerous studies had showed that MAPK pathway was involved to regulate the osteogenic differentiation of bone cells and MSCs by a Ras-dependent signal transduction pathway^21^,^22^. Therefore, to determine whether the cascade of classical MAPK pathway was activated following the induction of Mg alloys extracts at 5, 15, 30 and 60 min, the phosphorylation events were detected. As shown in Fig. 10A. The increase of relative amount of p-ERK (p-ERK1/2/ERK1/2) was noticed with time, and reached the peak value at around 30 min. Ratio of p-ERK1/2 ERK1/2 then declined with prolonged osteoinduction by Mg alloys, and eventually reached almost the same level of pre-osteogenic induction (0 min). However, no detectable activation of JNK and p38 pathway could be observed at 5 min, 15 min, 30 min and 60 min as shown in Fig. 10.

## Discussion

As we known that the addition of alloying elements could improve the microstructure, mechanical and degradation properties of Mg and its alloys^23^. However, some alloying elements have potential hazard to the human body because of the adverse pathophysiological and toxicological effects. Meanwhile, the osteogenesis performance of the Mg is difficult to satisfy the need of the fracture healing^24^. Our previous study developed Mg-1Ca alloy for the use as biodegradable materials within bone, which had good mechanical and degradation properties^18^,^25^. Sr, as the essential trace element in human body, had pronounced effects on stimulating bone formation and inhibiting osteoclast resorption in bone regeneration^16^,^26^. Thus, specific interest in this study is addition of Sr in Mg-1Ca alloy because of its established beneficial effects on microstructural grain refinement, biocompatibility and osteogenesis ability.

Earlier scientific studies have revealed that pure Mg has poor corrosion resistance in physiological environments and rapidly loses its mechanical strength rendering them not suitable for load-bearing implants for long-term use^27^. Grain refinement was a suitable tool to improve the corrosion resistance and increase the mechanical strength, as well as enhancing the osteoblasts activity^28^. Our alloys characterization results illustrated that increasing Sr addition could promote the refinement of grain size and cause coarser grain boundaries in the alloy matrix, indicating an increased amount of brittle Mg_17_Sr_2_ intermetallic phase distributed in the grain boundary, which was the main factor enhancing the mechanical and corrosion properties. It was known that the Mg_17_Sr_2_ was more stable than α-Mg and had a higher corrosion potential than α-Mg^29^. Meanwhile, Mg-1Ca-2.0Sr alloy possessed the highest protein adsorption among the four alloys. Since protein adsorption onto the surface is important for the integration of implants with the surrounding tissues^17^, also has an crucial influence on the response of cell attachment and spreading at the early stage^30^, better early adhesion may result in more cell proliferation and then excellent biocompatibility.

The process of bone formation involves three major phases: proliferation, extracellular matrix maturation and mineralization. Which are orchestrated by various key molecules that regulate this phase transfer^31^, including the production of ALP, processing of pro-collagen to collagen, and the deposition of extracellular matrix containing additional proteins which would later be mineralized^32^. In general, both of the ALP activity and collagen secretion were increased as osteoblasts differentiation especially at the early stage of osteogenesis, and eventually calcium was deposited at the later stage. Thus, ALP activity, collagen synthesis and mineralized nodules formation were the most recognisable biochemical markers of osteoblasts activity. Furthermore, Osteoblast differentiation is tightly controlled by a diverse set of internal and external factors such as growth factors, transcriptional factors and signaling pathways that result in the formation of mineralized bone^33^. RUNX2 is among the most important transcription factors necessary for early osteoblast differentiation^34^. It is reported that RUNX2 knockout mice show a lack of bone development and formation^35^. RUNX2 is also demonstrated to be responsible for the activation of osteoblast differentiation markers genes, including ALP, OCN, COL I and so on. For instance, RUNX2 triggers the ALP expression by the mechanism of binding to the ALP promoter region. OSX, as a downstream gene of RUNX2, is a novel zinc finger transcription factor and specifically required for the commitment of pre-osteoblasts to differentiate into mature osteoblasts^36^,^37^. ALP, as an early stage indicator for bone differentiation, is vital in the calcification of bone matrix. BSP appears following ALP expression and promotes the nucleation of hydroxyapatite mineralization, and then increases nodule formation^38^. COL I, as a major protein constituting the bone matrix, provides the backbone for the maturation and mineralization of the bone matrix. OCN is a late-stage marker of osteoblast differentiation, and its product denotes the onset of ECM deposition^39^. Our results suggested that Mg-Ca-Sr alloys could promote osteogenic differentiation and mineralization potentiality, especially Mg-1Ca-2.0Sr alloy. The molecular understanding of these results was further confirmed by the relative expression level of above molecular index via RT-PCR reaction (Fig. 9). However, it’s notable that OPN expression was down-regulated. Previous studies had described that OPN was a possible negative regulator of proliferation and differentiation in MC3T3-E1 cells^40^. Another evidence also suggested that OPN had resorptive activity of mature osteoclasts as a potent stimulator of the osteoclastogenesis^41^. Our results seem to be consistent with these studies, speculating that the OPN, as a suppressor of osteoblast differentiation, was inhibited by Mg-Ca-Sr alloys. The difference in above outcomes (osteogenesis effects and osteogenesis-related gene expression) may be due to the different degradation products of four Mg-Ca-Sr alloys in culture medium, including the concentration of Mg^2+^, Ca^2+^ and Sr^2+^, as well as the local microenvironment of pH. However, the underlying mechanisms and potential signaling pathways still need to be further studied.

The mitogen-activated protein kinases (MAPK) are the family of secondary messengers that convey signals from the cell surface to nucleus in response to a wide range of external stimuli such as biomaterials^42^,^43^. MAPK family involves in the regulation of many cellular physiological functions such as proliferation, differentiation, inflammation and apoptosis, and MAPKs are indispensable factors in osteoblast initiation process, especially the ERK/MAPK pathway^44^. It has been reported that inactivation of ERK in osteochondroprogentior cells caused a block in osteoblast differentiation and led to ectopic chondrogenic differentiation^45^. Many previous studies have discussed the relationship between MAPK family and RUNX2/OSX, the results indicated that ERK1/2 mediated RUNX2 phosphorylation and transcriptional activity in bone^46^. Consistent with these reports, our Western Blotting results of phosphorylated MAPKs showed significant ERK1/2 activation as well as the up-regulation of RUNX2 expression in osteoblasts induced by Mg-1Ca-2 Sr alloy without increases in total ERK level, however, there were no obvious differences in the amount of total and phosphorylated JNK or p38 between the treatment and control group (Fig. 10). ERK1/2 signaling pathway is activated by a variety of cell growth factors and then transmits extracellular signals from the cell surface to the nucleus, which transmission played important roles in the process of cell proliferation and differentiation^47^. Literatures have demonstrated that the individual Mg^2+^, Ca^2+^ or Sr^2+^ could all increase osteoblasts activity and induce osteogenic differentiation. Mg^2+^ could bind to the subunit of integrins and then up-regulated the expression of integrins in osteoblasts. Among the multiple integrins, α5β1 could selectively bind to fibronectin to engage the cells and successively activate the focal adhesion kinase (FAK)^48^. Available evidence indicated that FAK plays a crucial role in integrating integrin signals to direct ERK signaling activation, RUNX2 transcription, and then osteogenic differentiation of osteoblasts^49^. Ca^2+^ and Sr^2+^ could promote osteogenesis via binding to calcium-sensing receptor (CaSR). Once the CaSR is activated by the increased divalent cation, the intracellular signaling pathways start to activate different G proteins. This leads to the activation of tyrosine kinases, phospholipase C and adenylate cyclases, which trigger the phosphorylation-activation of ERK1/2^50^,^51^. Sr^2+^ and Ca^2+^ may act together to promote the bone formation in a concentration-dependent manner, producing an additive effect^52^. Thus we speculated that Mg-1Ca-2Sr alloy may regulate the Ras/Raf/MEK/ERK pathway through binding CaSR and integrins to activate Raf system and modulate ERK1/2 activation positively.

**Figure 11:**
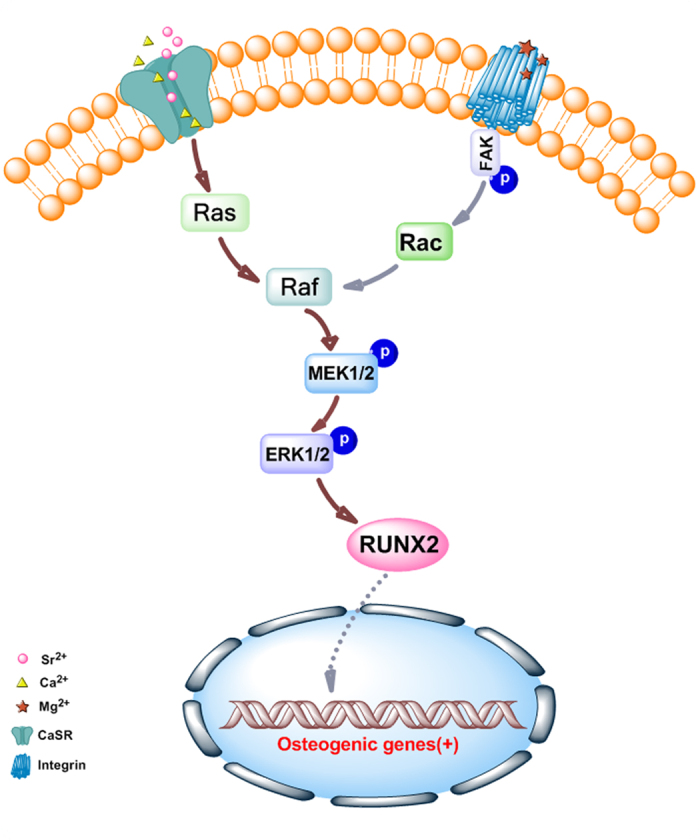
Schematic illustration of the general idea for this study and the possible signaling pathway of Mg-1Ca alloys with Sr contents. The alloys release Mg^2+^, Ca^2+^ and Sr^2+^. Mg^2+^ could bind to the subunit of integrins and then activate MEK/ERK pathway. Ca^2+^ and Sr^2+^ could promote osteogenic gene expression via binding to calcium-sensing receptor (CaSR) and activating ERK signaling pathway through Ras/Raf/MEK/ERK pathway.

Based on our present work, the Mg-1Ca-xwt.% Sr alloy extracts significantly increased the proliferation, viability as well as the osteogenesis differentiation of the MC3T3-E1 cells *in vitro*. We further found out that the Ras/Raf/MEK/ERK of MAPK signal pathway played a vital role in the differentiation and maturation of osteoblasts cultured with Mg-1Ca-2Sr alloy alloys. The general idea for this study was illustrated in Fig. 11. Our results suggested that the Mg-1Ca-2Sr alloy will be a potential candidate for orthopaedic implants. Furthermore, the *in vivo* tests for the future studies should also be applied to better understand the osteoinduction behavior of the alloys.

## Methods

### Mg Alloy fabrication and preparation

The Mg-Ca-Sr alloys with a nominal chemical composition of 1wt.% Ca and 0.2–2 wt.% Sr were prepared from commercial pure Mg (99.7%), Ca (99.8%), and Sr (99.9%). The raw materials were melted in a high purity graphite crucible under the protection of high purity Argon (99.99%) atmosphere at about 670 °C. After addition of the alloy elements, temperature of the melts was increased to 720 °C. Being held for 40 min, the melts were then poured into a steel mold preheated to 250 °C. The as-cast ingots of Mg-Ca-Sr alloys were treated with solid solution at 340 °C for about 4 h, followed by quenching in water. Finally, the alloy samples were hot extruded at about 320 °C with an extrution speed of 2 mm/min. Subsequently, the disk sample with a diameter of 12 mm and a height of 2 mm were cut from the extruded ingots and abraded with up to 2000-grit Sic water paper, and then ultrasonic cleaned.

### Characterization of alloys

The elementary compositions of the alloys were determined using inductively coupled plasma atomic emission spectrometry (ICP-AES). The samples for microstructural characterization were polished to 2000 grits and followed by etching in 4% nitric acid solution, and then investigated through optical microscope (Olympus BX51M) and field emission scanning electron microscopy (FESEM, Nova Nano SEM 430). Crystallographic phase identification was performed using X-ray diffraction (XRD, Rigaku DMAX 2400) and the chemical composition was analyzed by Energy dispersive spectrometer (EDS, Nova Nano SEM 430).

### Protein adsorption assay

A 1 mL droplet of α-MEM (Gibco) with 10% Fetal Bovine Serum (FBS, Gibco) was introduced onto each sample by a pipette. After incubation for 4 h at 37 °C, specimens were washed three times with phosphate buffered saline (PBS) and protein from samples was lysed in 0.2 vol. % Triton X-100 for 12 h at 4 °C. The concentrations of protein in the collected solutions were determined by a Micro-BCA protein assay kit (Pierce). Each experiment was carried out in triplicate.

### Hemolysis test

The Hemolysis test was approved by the Ethics Committee at Guangzhou General Hospital of Guangzhou Military Command, China. It is in accordance with National Statement on Ethical Conduct in Research Involving Humans and the informed consent was obtained from the subject. The existence of hemolysis factor in the biomaterials lead to the destruction of erythrocyte during the contact with human blood, as the dissociative hemoglobin increased, the toxicity of the materials to human body increased. Therefore, qualitative or quantitative analysis can be measured by absorption spectrometry. Hemolysis test was conducted according to DIN ISO 10993-4. Healthy human blood from a volunteer containing sodium citrate (3.8 wt.%) in the ratio of 9:1 was taken and diluted with normal saline (4:5 ratio by volume). Each Mg alloy specimen was soaked in 10 mL of normal saline that were preheated for 30 min at 37  °C. Then 0.2 mL of diluted blood was added and the mixtures were incubated for 60 min at 37  °C in thermostatic water bath. Finally, the tube was centrifuged at 3000 rpm for 5 min and 100 μL of supernatant was transferred to a 96-well plate. The optical density (OD) was measured at 545 nm. Normal saline and deionized water was used as the negative control and positive control, respectively. The hemolysis ratio (HR) was calculated based on the average of three replicates as:


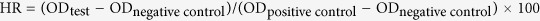


### Cells culture

The murine calvarial pre-osteoblast (MC3T3-E1) (ATCC, CRL-12424) was adopted and cultured in α-MEM medium containing 10% FBS at 37 °C in a humidified atmosphere of 5% CO_2_ routinely. For the indirect contact test with pre-osteoblasts, the extracts of Mg alloy samples were prepared using α-MEM medium containing 10% FBS with the surface area of extraction medium ratio 1.25 cm^2^/mL at 37 °C for 24 h. Osteogenic differentiation of MC3T3-E1 cells was induced by the different extracts of Mg alloys supplemented with osteogenesis revulsant (0.01 μM dexamethasone, 50 ug/mL ascorbic acid and 10 mM Na-β-glycerophosphate).

### Cell viability

CCK-8 was used as an index of cell viability in the culture media. Briefly, 100 μL of cell suspension were seeded in 96-well plate at a density of 2 × 10^4^ cells/mL and incubated for 24 h. The culture medium was then removed and replaced by different alloy extracts. α-MEM medium with 10% FBS was used as blank control. After co-incubation for 1, 4 and 7 days, cell viability was assessed using Cell Counting Kit-8 (CCK-8, DOJINDO) assay according to the manufacturer’s instructions. At the prescribed time points, cells were gently rinsed three times with PBS, and 10 μL CCK-8 was added into each well, followed by continuous incubation for 4 h. The OD value at 450 nm was measured in Microplate reader (Thermo, Multiskn Go). Each experiment was carried out in triplicate.

### Intracellular total protein and Alkaline phosphatase activity assay

100 μL of cell suspension was seeded in the 96-well plates at a density of 2 × 10^4^ cells/mL and cultured in different extracts supplemented with osteogenesis revulsant. At day 14 after passage every two days, the alkaline phosphatase (ALP) activities were determined by a colorimetric assay using an ALP reagent containing p-nitrophenyl phosphate (p-NPP) as the substrate, and the absorbance of p-nitrophenol (p-NP) formed was measured at 405 nm. The intracellular total protein content was determined using the Micro-BCA protein assay kit simultaneously, and the ALP activity was finally normalized to the total protein content correspondingly. Each experiment was carried out in triplicate.

### Collagen secretion

Collagen secretion was quantified by Sirius Red staining as the literature described^53^. In brief, cells were cultured in different extracts supplemented with osteogenesis revulsant. At day 14 after passage, cells were washed three times with PBS and fixed in 4% paraformaldehyde, and then stained for collagen secretion with a 0.1% solution of Sirius Red (Sigma) in saturated picric acid for 18 h. After washing with 0.1 M acetic acid until the red color disappeared, images were taken under inverted phase contrast microscope. For the quantitative analysis, the stain was eluted by the destain solution (0.2 M NaOH/methanol 1:1). The OD value at 540 nm was then measured using a microplate reader. Each experiment was carried out in triplicate.

### Extracellular matrix mineralization

Matrix mineralization by osteoblasts was evaluated by Alizarin Red staining. After culture with different extracts, cells were washed three times with PBS and fixed with 75% ethanol for 1 h, and then stained with 40 mM Alizarin Red S (Sigma, pH 8.3) for 30 min at room temperature. After washing with deionized water until no more color appearing, the staining was visualized by inverted phase contrast microscope and performed to analyze calcium deposits and then dissolved in 10% cetylpyridinium chloride in 10 mM sodium phosphate (pH = 7) and the OD value was measured at 620 nm for the quantitative analysis.

### Quantitative Real-time PCR

The expressions of osteogenesis-related genes and integrin subunits were evaluated by real-time polymerase chain reaction (RT-PCR). Cells were seeded with 2×10^4^ cells/well and cultured for 14 days. Total RNA of adherent cells was isolated using the TRIzol reagent (Invitrogen). 1 mg of RNA from each sample was reversed transcribed into complementary DNA (cDNA) using the PrimeScript^TM^ reagent kit (Takara). Expression level of genes including RUNX2, OSX, Integrin α5, Integrin β1, ALP, BSP, COL I, OCN and OPN were quantified using Rotor-gene Q (Qiagen) with SYBR^®^ Premix Ex^TM^ Taq II (TaKaRa). The cycling protocol were set as follows: 95 °C for 15 min, followed by 45 cycles including 95 °C 5 s and 60 °C 30 s. All reactions were carried out in triplicate and the RT-PCR results were analyzed using the Rotor-Gene Real-Time analysis software 6.0. In each case, the data were normalized to the expression level of GAPDH as a housekeeping gene^54^. The forward and reverse primers of the selected genes were listed in Table 3.

### Western Blot analysis

Adherent cells were washed three times at the indicated time with ice-cold PBS and then lysed using lysis buffer (Takara) on ice for 30 min. After being centrifuged at 12,000 rpm for 10 min at 4 °C, the protein concentrations were determined by the Micro-BCA protein assay kit. For Western blot analysis, 20 μg of each protein sample underwent 10% SDS-PAGE and electrotransfered onto PVDF membranes treated with 20% methanol in Tris-glycine buffer. After being blocked with PBS containing 5% bovine serum albumin (BSA) for 2 h at room temperature, blots were probed with 1:1000 diluted primary monoclonal (Cell Signaling Technology) including anti-mouse ERK1/2, phosphorylated (p-) ERK1/2, anti-mouse JNK, phosphorylated (p-) JNK, anti-mouse P38 and phosphorylated (p-) P38 at 4 °C overnight, and then with 1:2000 diluted horseradish peroxidase (HRP)-conjugated secondary antibody (Rockland Immunochemicals Inc) for 1 h at room temperature. Signals were then visualized by enhanced chemiluminescence detection reagents (Millipore) and developed with Kodak film.

### Statistics

Data were analyzed by SPSS 13.0. Statistical comparisons among groups were evaluated by One-way ANOVA and Post Hoc multiple comparison LSD. The results were presented as means ± standard deviation (SD). A p-value less than 0.05 and 0.01 was considered statistically significant and highly significant, respectively.

## Conclusions

In this study, we developed ternary Mg-1Ca-xwt.% Sr (x = 0.2, 0.5, 1.0, 2.0) alloys for potential application as orthopedic biodegradable implant materials. The results of present study showed that Mg-1Ca alloys were grain refined with different levels of Sr added during the melting process. The resultant cast alloys could stimulate cell viability and osteogenic differentiation. The degree of stimulation varied depending on the Sr content. Our findings finally demonstrated that a 2.0 wt.% Sr addition in Mg-1Ca alloy induced higher protein adsorption than the other three alloys, as well as led to the best cell functions including proliferation, and osteogenic differentiation represented by ALP activity, collagen secretion, extracellular mineralization and osteogenesis-related genes expressions through ERK1/2 pathway. In summary, all these results suggest that the Mg-1Ca-2.0Sr alloy possessed a unique osteogenic stimulatory ability and may be used as a biomaterial for bone regeneration and tissue engineering applications.

## Additional Information

**How to cite this article**: Li, M. *et al.* Stimulatory effects of the degradation products from Mg-Ca-Sr alloy on the osteogenesis through regulating ERK signaling pathway. *Sci. Rep.* **6**, 32323; doi: 10.1038/srep32323 (2016).

## Figures and Tables

**Table 1:** The actual chemical composition of the alloys by ICP-AES.

Alloy	Chemical composition (wt.%)		
	Mg	Ca	Sr
Mg-1Ca-0.2Sr	98.75	1.01	0.24
Mg-1Ca-0.5Sr	98.38	1.13	0.49
Mg-1Ca-1.0Sr	98.05	0.96	0.99
Mg-1Ca-2.0Sr	96.89	1.04	2.07

**Table 2:** Hemolytic ratio (HR) of the ternary Mg-1Ca-xwt.% Sr alloy samples.

Alloys	Mg-1Ca -0.2Sr	Mg-1Ca -0.5Sr	Mg-1Ca -1.0Sr	Mg-1Ca 2.0Sr	Negative group	Positive group
HR (%)	0.21	1.48	1.05	2.01	—	—

**Table 3:** Primers sequence used for Real-time PCR.

Gene	Primer sequence	
	Forward	Reverse
**RUNX2**	5′-TCCAACCCACGAATGCACTA-3′	5′-GAAGGGTCCACTCTGGCTTTG-3′
**OSX**	5′-AGAAGCCATACGCTGACCTTTC-3′	5′-AGTGAGGGAAGGGTGGGTAGTC-3′
**Integrinα5**	5′-AGCAACTGCACCTCCAACTACA-3′	5′-ACACTTGGCTTCAGGGCATT-3′
**Integrinβ1**	5′-TTGGTCAGCAACGCATATCTG-3′	5′-CAGCAAAGTGAAACCCAGCAT-3′
**ALP**	5′-GTCATCATGTTCCTGGGAGA-3′	5′-GGCCCAGCGCAGGAT-3′
**BSP**	5′-AATGGCCTGTGCTTTCTCGAT-3′	5′-CCCTGGACTGGAAACCGTTT-3′
**COL I**	5′-GCAGGGTTCCAACGATGTTG-3′	5′-AGGAACGGCAGGCGAGAT-3′
**OCN**	5′-CAATAAGGTAGTGAACAGAC-3′	5′-CTTCAAGCCATACTGGTCT-3′
**OPN**	5′-TCCAAAGCCAGCCTGGAAC-3′	5′-TGACCTCAGAAGATGAACTC-3′
**GAPDH**	5′-CATGGCCTTCCGTGTTCCTA-3′	5′-CCTGCTTCACCACCTTCTTGAT-3′
